# A *G542X* cystic fibrosis mouse model for examining nonsense mutation directed therapies

**DOI:** 10.1371/journal.pone.0199573

**Published:** 2018-06-20

**Authors:** Daniel R. McHugh, Miarasa S. Steele, Dana M. Valerio, Alexander Miron, Rachel J. Mann, David F. LePage, Ronald A. Conlon, Calvin U. Cotton, Mitchell L. Drumm, Craig A. Hodges

**Affiliations:** 1 Department of Genetics and Genome Sciences, Case Western Reserve University, Cleveland, Ohio, United States of America; 2 Department of Pediatrics, Case Western Reserve University, Cleveland, Ohio, United States of America; 3 Department of Physiology and Biophysics, Case Western Reserve University, Cleveland, Ohio, United States of America; University of Florida, UNITED STATES

## Abstract

Nonsense mutations are present in 10% of patients with CF, produce a premature termination codon in *CFTR* mRNA causing early termination of translation, and lead to lack of CFTR function. There are no currently available animal models which contain a nonsense mutation in the endogenous *Cftr* locus that can be utilized to test nonsense mutation therapies. In this study, we create a CF mouse model carrying the *G542X* nonsense mutation in *Cftr* using CRISPR/Cas9 gene editing. The *G542X* mouse model has reduced *Cftr* mRNA levels, demonstrates absence of CFTR function, and displays characteristic manifestations of CF mice such as reduced growth and intestinal obstruction. Importantly, CFTR restoration is observed in *G542X* intestinal organoids treated with G418, an aminoglycoside with translational readthrough capabilities. The *G542X* mouse model provides an invaluable resource for the identification of potential therapies of CF nonsense mutations as well as the assessment of *in vivo* effectiveness of these potential therapies targeting nonsense mutations.

## Introduction

Cystic Fibrosis (CF) is an autosomal recessive genetic disorder caused by mutations in the cystic fibrosis transmembrane conductance regulator (CFTR) gene. CFTR is an anion channel expressed throughout the body with highest expression in epithelial tissues. Absence of CFTR function impairs transepithelial fluid and electrolyte permeation and results in viscous mucus along the epithelial lining of organs leading to a wide range of disease manifestations. Common manifestations of CF include lung failure [1–3], pancreatic insufficiency [4], intestinal obstruction [5–8], and reduced growth [9–11]. Over 2000 unique CFTR variants have been identified, with ~300 categorized as definitively CF-causing mutations [12, 13]. CF-causing nonsense mutations are the second most common CF mutation type, and are found in approximately 10% of patients with CF [14, 15]. Nonsense mutations create a premature termination codon (PTC) in *CFTR*, which leads to premature termination of CFTR translation [16].

Recently, there has been progress in the development of CFTR modulators that restore CFTR function in patients with specific CFTR mutations. Examples include VX-770 (ivacaftor) for gating mutations like *G551D* [17, 18] and VX-809 (lumacaftor) for the misfolding mutation F508del [19, 20]. However, no therapies are available to CF patients that restore function to nonsense mutations in *CFTR*. Certain drugs, like gentamicin and PTC124 (ataluren), have been shown to interact with the ribosome to induce readthrough of PTCs by allowing insertion of a near-cognate aminoacyl tRNA and translation of a full-length protein [21–23]. Both gentamicin and PTC124 have been used in clinical trials for patients with nonsense mutations [24–27]. However, gentamicin treatment is associated with nephrotoxicity and ototoxicity [28] and PTC124 has produced no significant improvements in adult CF patients with nonsense mutations in Phase 3 clinical trials [29]. Therefore, no currently available treatment for CF nonsense mutations is both safe and clinically effective.

Because of the safety issues surrounding PTC readthrough strategies, animal models of *CFTR* nonsense mutations are essential for *in vivo* validation of new therapies. Current CF mouse models with nonsense mutations were created in a way that either does not allow for correction of the nonsense mutation or [30, 31] or utilizes non-endogenous *CFTR* with tissue specific expression [14]. Therefore, a model with global expression of the *G542X* mutation in the endogenous mouse *Cftr* will have a greater value for examining the efficacy of nonsense mutation therapies on *Cftr* in multiple tissue types with native expression levels. In this manuscript, we describe the generation of a mouse model with expression of the *G542X* mutation, the most common nonsense mutation in CF [32], using CRISPR/Cas9 gene editing technology. Mice homozygous for the *G542X* mutation have reduced *Cftr* expression and absence of CFTR function in the airway and intestine. These mice display typical cystic fibrosis manifestations such as poor growth and reduced survival due to intestinal obstruction. Importantly, we demonstrate that pharmacological readthrough of the *G542X* nonsense mutation in this model allows production of functional CFTR. The *G542X* mouse will be a valuable model for the examination of nonsense mutation therapies for CF and other genetic diseases caused by nonsense mutations.

## Materials and methods

### Generation of the G542X allele

To produce the *G542X* mouse *Cftr* allele (*Cftr*^*G542X*^) using the gene editing system CRISPR/Cas9, guide RNAs (gRNA) were selected in exon 12 of mouse *Cftr* using CRISPR design software that identifies optimal gRNAs based on proximity to target as well as off-target cutting predictions [33]. Four of these gRNAs were tested in vitro using guide-it gRNA in vitro transcription and screening kit (Clontech) for the ability to guide Cas9 nuclease activity to the desired DNA sequence. One gRNA (25 ng/ul; PNABio), a 120 bp single stranded oligonucleotide (ssODN) containing the *G542X* mutation (25 ng/ul; IDT) centered on the cut site and either Cas9 mRNA (25–50 ng/ul; PNABio) or Cas9 protein (25 ng/ul; PNABio) were injected in the pronucleus of C57BL/6 one-cell embryos. Embryos were then placed in pseudo-pregnant females to develop. Ear punches from the 22 resulting pups were sequenced using next generation sequencing. The region of interest was PCR amplified (~300 bp) with flanking primers that were tailed with a universal sequence. A secondary PCR amplification was then performed on the primary PCR product using a set of primers specific for the universal sequence and tailed with I5,I7 Illumina index and specific barcode sequences allowing for the sequencing of multiple samples at the same time. The mixture of barcoded PCR products were analyzed on an Illumina MiSeq instrument using a paired-end 300 base pair kit allowing for 25 million reads per run. Data were de-convoluted and variant analysis in comparison to the original sequence was performed using the Outknocker software program [34].

### Mice

In some experiments, the *G542X* mouse model was compared to a previously published CFTR null model carrying a *S489X* mutation (*Cftr*^*tm1Unc*^) which is congenic on the C57BL/6J background [31]. Mice homozygous for these mutations were created by breeding heterozygous males and females and wild type littermates were used as controls. Genotyping was completed by PCR analysis using DNA extracts from ear biopsies. To detect the *G542X* allele (319 bp) primers P1 (5’- ACAAGACAACACAGTTCTCT -3’) and P2 (5’ TCCATGCACCATAACAACAAGT -3’) were used. To detect the wildtype (WT) allele (319 bp) P2 and P3 (5’- ACAAGACAACACAGTTCTTG -3’) were used in a separate reaction. PCR reactions were completed for 40 cycles of 95°C for 30 seconds, 58°C for 30 seconds and 72°C for 30 seconds and products were run out on 2% agarose gels. All mice were allowed unrestricted access to water and solid chow (Harlan Teklad 7960; Harlan Teklad Global Diets). All animals were maintained on a 12-h light, 12-h dark schedule at a mean ambient temperature of 22°C and were housed in standard polysulfone microisolator cages in ventilated units with corncob bedding. Animals were monitored on a daily basis, and weight was assessed every 5 days from 10 to 40 days of age. Length of 6-wk-old euthanized mice was assessed from nose to anus by use of digital calipers. For G418 treatment, mice were intraperitoneal injected on three consecutive days (25mg/kg b.w.). Twenty four hours after last injection, mice were sacrificed and lungs were flash frozen and RNA prepared as described below. The Institutional Animal Care and Use Committee of Case Western Reserve University approved all animal protocols.

### Expression analysis

One μg of RNA was reversed transcribed in cDNA using QScript cDNA synthesis kit (VWR). Real-time quantitative PCR was performed on a StepOne PCR system (Applied Biosystems). *Cftr* expression was assessed using a TaqMan expression assay which used primers spanning exon 17 and 18 (Mm00445197; Applied Biosystems). Expression was normalized to β-actin as the endogenous control. Each RNA sample was used to make cDNA in duplicate and the expression results were then averaged to yield the final value. The average of each sample was then expressed as a percentage of wildtype expression.

### Bioelectric measurements

Nasal potential difference (NPD) measurements were obtained as previously described [35, 36]. NPD (mV) was assessed after the addition of chloride-free HEPES-buffered saline containing 10 μM forskolin. Short circuit measurements on intestinal sections were obtained as previously described [36]. The change in short-circuit current was calculated after the addition of 10 μM forskolin and 100 μM IBMX to the basolateral side of the intestinal sections.

### Intestinal organoid harvesting and culture

Intestinal organoids were harvested from adult mice as described by others [37]. Briefly, the mouse was sacrificed, the small intestine removed and flushed using PBS. The intestine was cut longitudinally, villi removed with a razor blade and the remaining intestine was cut into ~0.5 cm segments and suspended in Gentle Cell Dissociation Reagent (STEMCELL Technologies) in a 50 mL conical tube for 30 minutes under gentle agitation with a shaker. In a sterile tissue culture hood, the intestinal segments were vigorously shaken by hand for 30 seconds, and the supernatant was deposited in a 10 cm dish. The segments were resuspended in ice-cold PBS lacking Mg^++^ and Ca^++^, and vigorously shaken again for 30 seconds. This process was repeated until four fractions of supernatant were produced. The fraction which was most enriched for intestinal crypts was filtered using a 100 μm cell strainer, and pelleted at 450xg for 10 minutes. The supernatant was discarded, and the pellet was resuspended in 500 μl of a 1:1 mixture of MatriGel (Corning) and Intesticult Organoid Growth Media (OGM; STEMCELL Technologies). Crypts were diluted to a concentration of 10 crypts/μl, and plated into non-tissue culture treated 24-well plates, with 35 μl of MatriGel:OGM mixture deposited in each well. The MatriGel:OGM was solidified at 37°C for 15 minutes, then 500 μl of OGM was gently deposited in each well. The organoids were stored in a 37°C incubator with 5% CO_2_. The OGM was changed once every three days, and the organoids were passaged once every 7 days.

### Measurement of intestinal organoid swelling

Forskolin-induced swelling (FIS) of intestinal organoids was performed similar to previously described methods [38, 39]. Briefly, intestinal organoids which had been cultured for 6–8 days were split into non-tissue culture treated 48-well plates (Genesee Scientific) and allowed to grow for 24 hours before drug treatment. Organoids were treated with G418 (20–100 μM) or PTC124 (1–20 μM) for 72 hours prior to FIS measurements. FIS was assessed by treating organoids with 10 μM forskolin and imaging for 5 hours under live cell conditions with brightfield microscopy on a Lionheart FX Automated Microscope (BioTek). FIS was quantified by identifying and normalizing organoid area to T = 0 with Gen5 ImagePrime software (BioTek). Area under the curve (AUC) at T = 300 minutes was calculated to compare statistical significance between treatment groups. All organoids used for swelling quantifications were between passages 1–4.

#### Statistics

Results are expressed as the mean +/- S.E.M. Differences between groups were determined using either an ANOVA with post-hoc Tukey test or an unpaired t test. A P value of <0.05 was considered significant.

## Results

### Generation of the G542X mutation

**Fig 1:**
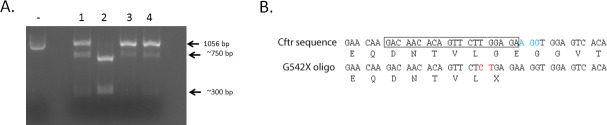
Generation of the *G542X* mutation. (A) To validate gRNA efficiency in guiding Cas9 nuclease to the desired site, an in vitro assay was performed. PCR amplified DNA containing exon 12 and surrounding region of mouse *Cftr* (1056 bp) is displayed on an agarose gel with no gRNAs (-) or with 1 of 4 different gRNAs (1–4). Cas9 nuclease activity results in the cleavage of the DNA into fragments of ~750 bp and ~300 bp. (B) Normal *Cftr* mouse DNA and amino acid sequence around the desired mutation site is shown with the gRNA sequence (in box) and the protospacer adjacent motif sequence recognized by Cas9 (in blue). A portion of the sequence for the *G542X* oligo is also shown with the substitution change shown in red. The G to T mutation corresponds to the glycine to stop mutation. A silent T to C mutation was also inserted to assist with genotyping and verify HDR had occurred.

To generate the *G542X* mutation in mouse *Cftr*, the CRISPR/Cas9 gene editing system was utilized. Guide RNAs (gRNAs) in proximity to the DNA sequence corresponding to glycine at position 542 in mouse *Cftr* exon 12 were selected and tested in vitro for their ability to support efficient Cas9 nuclease activity to the DNA region as indicated by the reduction of full length DNA amplicon (Fig 1A). The gRNA that supported the highest Cas9 nuclease activity was injected in one-cell mouse embryos along with a ssODN containing the *G542X* mutation and either Cas9 mRNA or protein (Fig 1B). DNA from all mice originating from these injected embryos was sequenced. Of 22 mice, 9 (40.9%) had at least one *Cftr* allele containing the *G542X* mutation due to homology-directed repair (HDR) with the ssODN, 5 (22.7%) did not have the G542X mutation but had other mutations (insertions/deletions) due to nonhomologous end joining and 8 (34.4%) had no mutation of *Cftr* in exon 12. One of the founder mice containing the *G542X* allele was selected to establish and expand the *G542X* colony.

### *Cftr* expression is reduced and CFTR function is absent in G542X mice

**Fig 2:**
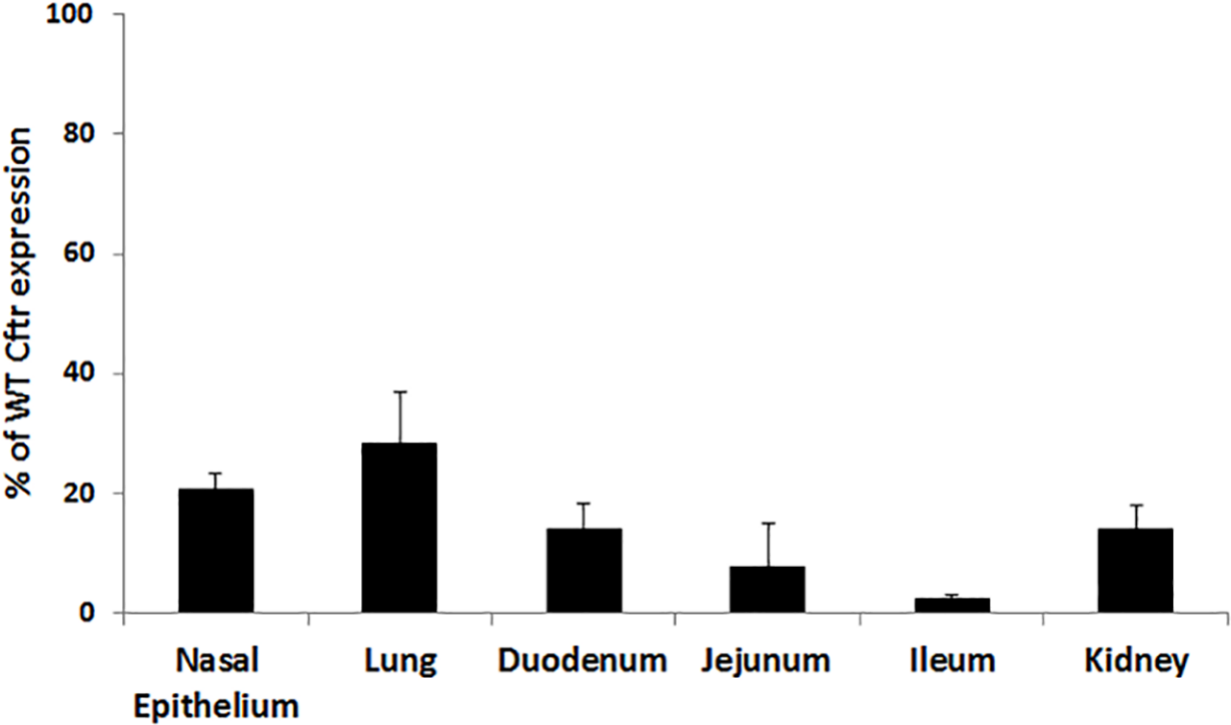
*Cftr* expression in tissues from *G542X* mice. *Cftr* expression in tissues from *G542X* and WT littermates were evaluated using qRTPCR. Airway (nasal epithelium, lung), intestine (duodenum, jejunum, ileum), and kidney *Cftr* expression as a percentage of WT *Cftr* expression is displayed. G542X expression was significantly reduced in all tissues compared to WT. (P<0.05 vs. WT. n≥5 per group).

**Fig 3:**
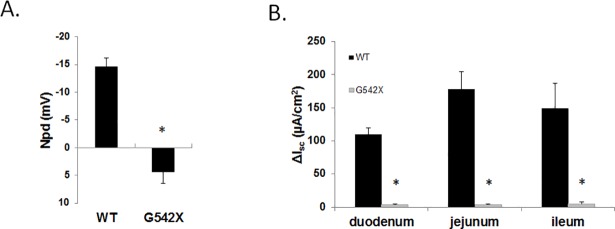
CFTR function in the airway and intestine of *G542X* mice. (A) NPD measurements and (B) change in intestinal short circuit current (ΔI_sc_) measurements from the duodenum, jejunum, and ileum from WT and *G542X* mice are shown. (*P<0.05 vs. WT. n≥4 per group).

*Cftr* expression was assessed using qRTPCR from various tissues of *G542X* and WT littermates. Cftr expression levels in tissues from *G542X* mice were significantly reduced compared to WT expression levels ranging from 2.5±0.5% in the ileum to 28.4±8.6% of WT levels in the lung (Fig 2). Assessment of CFTR function in the airway was performed using NPD measurements in the nasal lumen. Activation of CFTR and the subsequent Cl^-^ secretion into the nasal lumen causes a decrease in voltage across the nasal epithelium. Mice with functional CFTR display a decrease in NPD (-14.6±1.6 mV) while *G542X* mice have a slightly positive NPD (4.38±2.1 mV; Fig 3A) indicating no functional CFTR similar to other CF mouse models [35, 36, 40, 41]. CFTR function in the intestinal epithelium was examined by measuring short-circuit current (ΔI_sc_) in intestinal segments from *G542X* and WT mice before and after activation of CFTR. In WT intestinal segments, a significant increase in short-circuit current is observed following CFTR stimulation. Corresponding segments from *G542X* mice displayed significantly reduced changes in short-circuit current (Fig 3B), similar to other CF mouse models [36, 42, 43].

### G542X mice display characteristic CF manifestations

**Fig 4:**
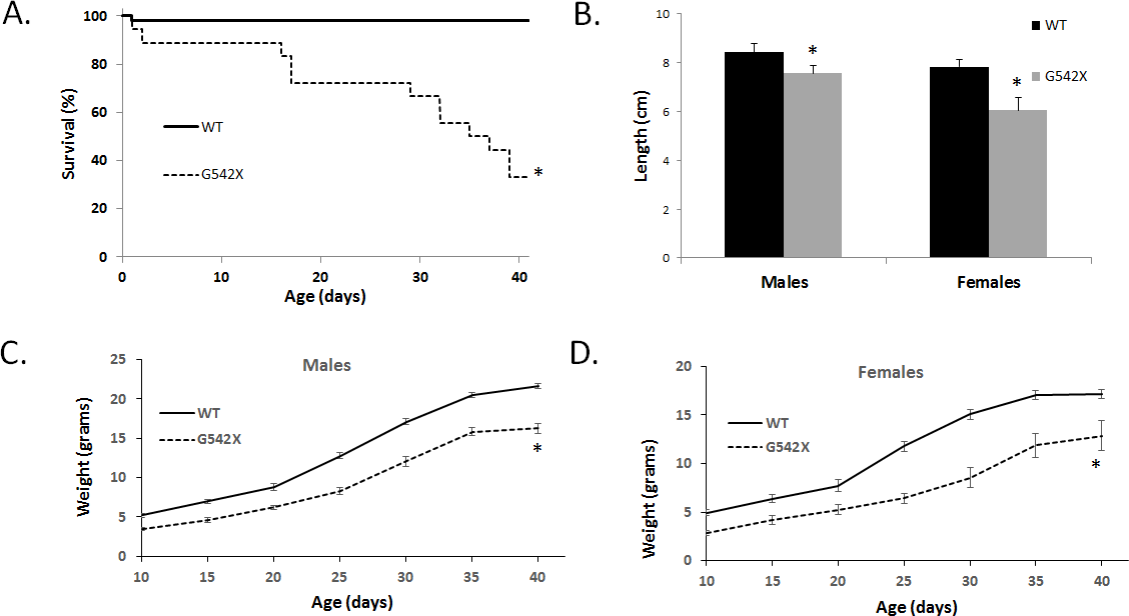
Survival and growth characteristics of *G542X* mice. (A) Survival of *G542X* and WT mice up to 40 days of age. (B) Length of *G542X* and WT mice at 6 weeks of age. (C, D) Weight of *G542X* and WT mice sex-matched mice up to 40 days of age. Weight at every age was significantly different between *G542X* and WT littermates. (n≥10 per group. *P<0.05 vs. WT).

The most common cause of morbidity in CF mouse models is intestinal obstruction [5, 36, 44, 45]. *G542X* homozygous mice displayed a reduced survival rate compared to WT littermates (33.3% vs. 97.9%; Fig 4A). Intestinal obstruction was observed in all *G542X* mice post-mortem but not in WT littermates. Another common disease manifestation in CF mouse models is reduced growth, or failure to thrive [31, 36, 43, 46]. *G542X* homozygous mice demonstrated significantly reduced length compared to sex-matched 6-week old WT littermates (in cm: males 7.6±0.2 vs. 8.4±0.1; females 6.0±0.3 vs. 7.8±0.1; Fig 4B). To assess growth, offspring from *G542X* heterozygote crosses were weighed at 5 day intervals from 10–40 days of age. *G542X* homozygous mice displayed significantly reduced weight at all time points compared to sex-matched WT littermates (Fig 4C and 4D).

### G542X CFTR function is restored following pharmacological nonsense mutation readthrough by G418 but not by PTC124

**Fig 5:**
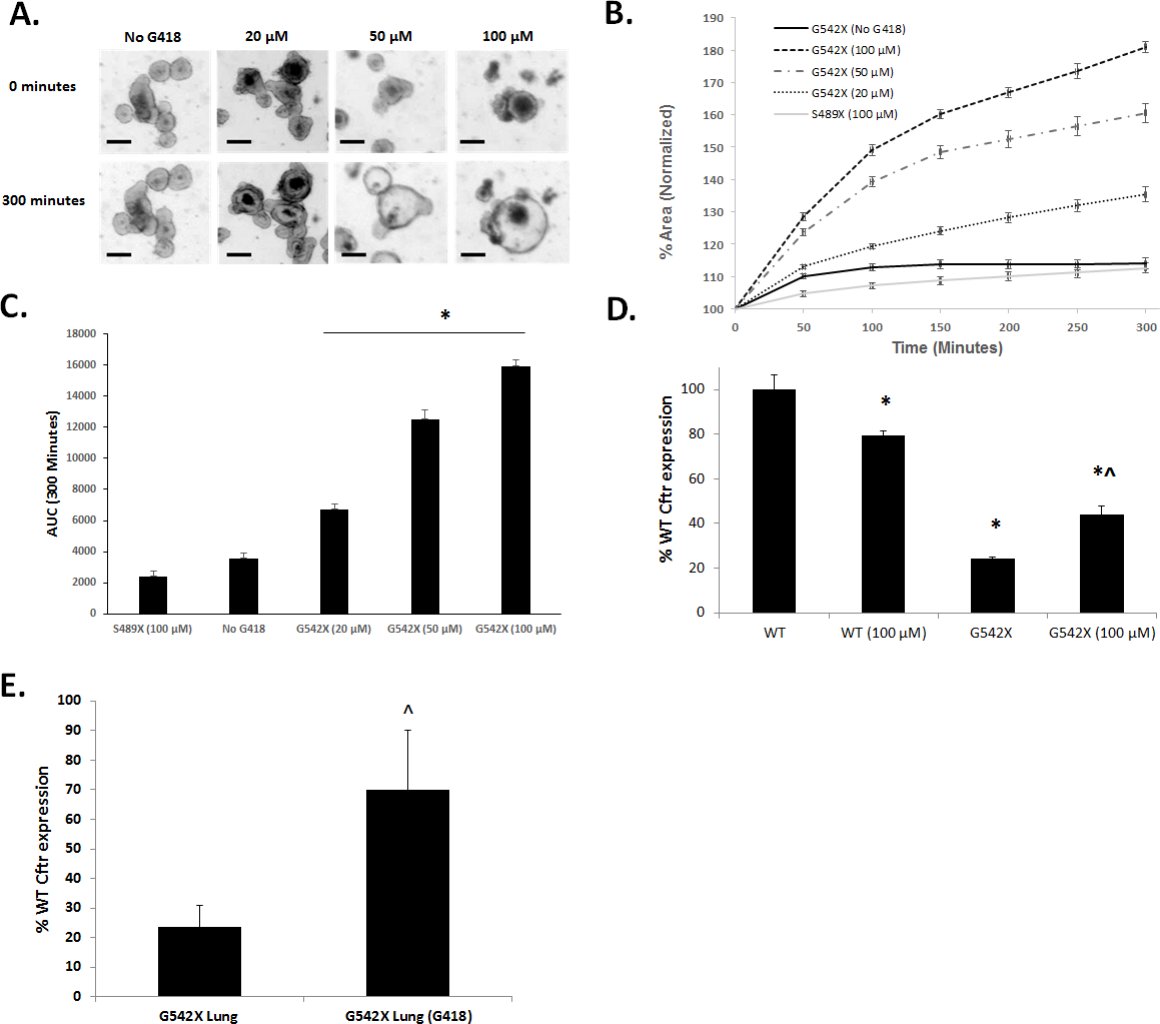
Intestinal organoids and tissue from *G542X* mice are used to test G418-mediated nonsense mutation readthrough. Intestinal organoids were incubated with indicated doses of G418, or vehicle for 72 hours prior to swelling with 10 μM forskolin. (A) Representative images at T = 0 and T = 300 minutes of organoid swelling, conditions as indicated. (Scale bar = 100 μm) (B) Total change in area was measured over 300 minutes, and FIS was quantified by normalizing the total organoid area to T = 0. (n = 5 wells of organoids for each treatment, ± SE). (C) AUC at T = 300 for indicated treatment groups. (*P<0.05 compared to untreated *G542X*). (D) Expression of *Cftr* in WT and *G542X* organoids with and without G418 treatment as a percentage of untreated WT organoids. (E) Expression of *Cftr* in lung from untreated *G542X* mice or treated with G418 compared to expression from WT mice. (*P<0.05 compared to untreated WT. ^P<0.05 compared to untreated *G542X*.).

**Fig 6:**
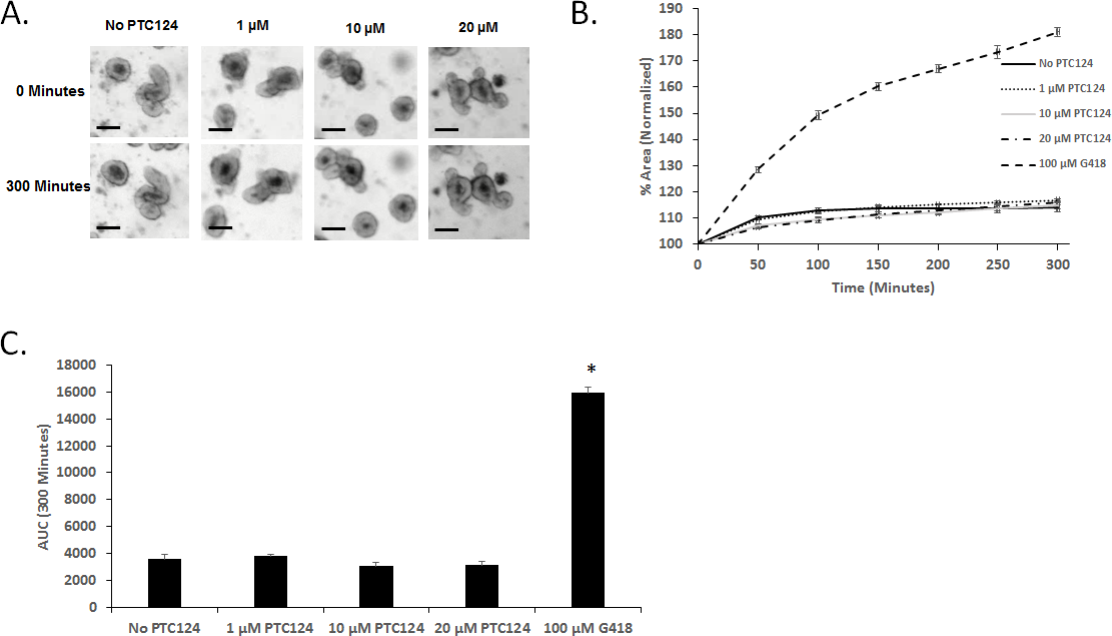
Intestinal organoids from *G542X* mice are used to test PTC124 mediated readthrough. G524X organoids were treated with the indicated doses of PTC124 for 72 hours prior to the FIS assay. (A) Representative images at T = 0 and T = 300 minutes of organoid swelling, conditions as indicated. (Scale bar = 100 μm). (B) Normalized areas for PTC124 treated organoids are shown, compared to vehicle and 100 μM G418-treated organoids. (C) AUC measurements at 300 minutes. (*P<0.05 compared to untreated *G542X*.).

The ability to produce functional CFTR following treatment with potential therapies is crucial for the *G542X* mouse model to have full utility as a model of CF nonsense mutations. To examine CFTR function following readthrough, we generated intestinal organoids from *G542X* homozygous mice. Intestinal organoids are a three-dimensional cell culture model produced from LGR5+ stem cells in the intestinal crypt with budding outgrowths and a hollow lumen [39]. Activation of CFTR by forskolin allows flow of Cl^-^ anions into the intestinal organoid lumen, increasing lumen osmolality, drawing in fluid, and causing organoid FIS. FIS is absent in organoids lacking CFTR function, allowing sensitive detection of CFTR activity. *G542X* organoids were treated for 72 hours with the aminoglycoside G418, a suppressor of nonsense mutations [47, 48]. FIS was examined by imaging organoid swelling over 300 minutes under live cell conditions with brightfield microscopy (Fig 5A). We observed a dose-dependent FIS response to G418-induced readthrough indicating restored CFTR function (normalized percent area at 300 minutes: No G418: 114±1.7; 20 μM: 135±2.3; 50 μM: 160±3.0; 100 μM: 181±1.7, Fig 5B). Statistical significance between treatment groups was compared by calculating area under the curve (AUC) at 300 minutes for each treatment groups (Fig 5C). AUC of organoids treated with G418 were significantly increased compared to non-treated organoids (Fig 5C). S489X organoids, which are non-correctable *Cftr*-null mutants, did not display FIS following 72 hour treatment with 100 μM G418, confirming that FIS in *G542X* organoids is due to readthrough of nonsense mutations (Normalized percent area at 300 minutes: 113±0.7, Fig 5B and 5C). Similar to tissues from *G542X* homozygous mice, cultured intestinal organoids have a reduced amount of *Cftr* expression compared to those from WT littermates (24.0±1.0%) (Fig 5D). However, concordant with the increase in CFTR function in G418 treated organoids from *G542X* mice, G418 treatment also significantly increased the amount of detectable *Cftr* mRNA (Fig 5D). Short term treatment of *G542X* mice with G418 also resulted in a significant increase in the amount of detectable *Cftr* mRNA in tissue (Fig 5E). Interestingly, PTC124 treatment of the *G542X* organoid model did not stimulate detectable FIS at any of the concentrations tested and thus resulted in no significant difference of AUC between PTC124 treated and non-treated organoids (1–20 μM; Fig 6A–6C).

## Discussion

Currently there are only two FDA approved therapies that directly target CFTR dysfunction, the primary defect of CF [17, 20]. These therapies are approved to modulate aberrant protein that is produced from the *CFTR* gene in patients with specific mutations that do not include nonsense mutations. Nonsense mutations typically result in little to no protein function. Reduced mRNA levels, through nonsense-mediated decay (NMD), dramatically decreases the amount of protein produced. In addition, any protein that is synthesized is truncated due to the PTC and, depending on severity of the truncation, leads to significantly limited protein function [49, 50]. Current research efforts to increase the amount of full length protein from nonsense mutations include both inducing readthrough of the PTC and targeting the NMD pathway to increase mRNA levels [49, 50].

Readthrough of PTCs through nonsense suppression can be achieved with aminoglycosides that reduce the efficiency of termination and allow for the insertion of near-cognate tRNA [21]. Promising results in cell and mouse models of nonsense mutations have led to the use of the aminoglycoside gentamicin in patients [14, 21, 24, 51]. In CF, functional improvements in CFTR have been described in CF patients receiving topical application to the nasal mucosa [26] but renal and otic toxicities are a concern with long term treatment. Interestingly, a minor component B1 of gentamicin has been shown to provide effective readthrough activity [52], suggesting that an enrichment of this component of gentamicin or modified aminoglycosides with reduced toxicity may hold promise for the future [23]. A high-throughput screen identified PTC124 as a candidate compound with readthrough ability without the toxicity concerns of aminoglycosides [22]. Despite a lack of readthrough efficacy of PTC124 in specific in vitro reporter systems [53], PTC124 treatment of preclinical models of CF have displayed functional improvements [54]. While some initial patient studies suggested PTC124 efficacy in CF [25, 55], larger studies failed to show significant improvements in CF patients with nonsense mutations in Phase 3 clinical trials [29]. While PTC124 will not be used as a therapy for CF, investigation of the compound is still underway for other nonsense mutation genetic disorders such as Duchenne muscular dystrophy.

There has been recent success in the identification of possible nonsense mutation suppressors in CFTR using high-throughput screens [56–59]. The *in vitro* tools created in each of these screens made the identification of these hits possible. However, a CF animal model to carry out *in vivo* studies to test the efficacy of these hits is not available to the research community. The first *Cftr* mutation made in the mouse was a nonsense mutation created by replacing the normal exon sequence with the premature stop codon, S489X along with selectable markers. The integration of the selectable markers in the exon precludes the production of functional protein [30, 31] and thus serves as a negative control for readthrough strategies. The only current animal model shown to produce functional CFTR following readthrough is a transgenic mouse that expresses human CFTR cDNA containing the *G542X* mutation driven by a rat Fatty Acid Binding Protein (FABP) promoter that results in intestinal-specific expression [14]. The conditions of *CFTR* expression in this model differ significantly from endogenous expression and readthrough of G542X can only be assessed in the intestine. In addition, because cDNA was used and there is no intron splicing, the mRNA produced is not susceptible to NMD which limits the interpretation of any *in vivo* studies. Non-endogenous expression levels and/or the absence of NMD of the resulting mRNA may explain why PTC124 displayed functional improvement in the intestine of hCFTR-G542X [54] model but failed to show similar improvements in patients.

In this study, we created a mouse model of CF containing the *G542X* mutation in the endogenous *Cftr* gene. We utilized the CRISPR/Cas9 gene editing system to create the *G542X* mutation in one-cell mouse embryos. This is the first report of *Cftr* CRISPR/Cas9 editing to create a mouse model. As previously reported, the use of CRISPR/Cas9 significantly reduces the time of gene editing in embryos compared to embryonic stem cells [60]. In addition, this gene editing system is highly efficient in the creation of the desired mutation. Traditional gene editing in embryonic stem (ES) cells typically produced 1–5% of ES colonies harboring the correctly edited gene and could take 1–2 years to produce the targeting construct and correctly targeted founder mice. With CRISPR/Cas9, 40.9% of founder mice contained the *G542X* mutation and were produced in as few as 3 months. One disadvantage in utilizing CRISPR/Cas9 gene editing is the possible creation of double-strand breaks in similar sequences to the target which can create off-target effects [61]. A low incidence of off-target mutations has been identified in mice created using CRISPR/Cas9 [62, 63]. While off-target mutations can occur, subsequent backcrosses to a parental strain (e.g., C57Bl/6) can remove any unintended consequences from off-target mutations.

Mice homozygous for the *G542X* mutation demonstrate reduced *Cftr* expression throughout the body. While our analysis cannot rule out that a truncated form of CFTR protein is produced, there is a clear absence of CFTR function in the airway and intestine of these mice. The *G542X* mice also display severe CF manifestations, including reduced growth, and reduced survival due to intestinal obstruction. In this model, mRNA levels can be increased and CFTR function can be corrected through readthrough by G418. Interestingly, the utilization of PTC124 in the intestinal organoid model did not demonstrate any improvement in CFTR function. This is consistent with studies that show no significant improvement in CF patients chronically administered PTC124. All of these findings confirm that this *G542X* mouse model can be utilized to test the efficacy of nonsense mutation therapies.

The *G542X* mouse model also provides an unlimited source of primary cells to potentially identify new therapies for nonsense mutations. For example, the prominent intestinal organoid FIS phenotype, which indicates CFTR function, has the potential to be used for high-throughput screens similar to those currently utilizing CF patient samples [39, 64]. The high degree of similarity between organoids and tissue *in vivo* may increase the odds of identifying translationally beneficial compounds in a high-throughput screen compared to current screens using transfected cell lines or *in vitro* reporter assays.

In conclusion, this is the first study to generate a mouse model containing CRISPR/Cas9 edited *Cftr* and the first CF animal model that can be utilized to assess whole body efficacy of nonsense mutation directed therapies. With several recent screens identifying potential PTC readthrough compounds, the *G542X* mouse model can be utilized to prioritize which compounds to test in a clinical trial that may be beneficial to CF patients. The ability to analyze *in vivo* effectiveness of current and future potential nonsense mutation therapies will not only increase our understanding of how these therapies work on a basic level but lead to treatments for CF patients with nonsense mutations.
